# Prevalence, associated factors and etiologic agents of oral candidiasis among HIV-positive patients in a Vietnamese general hospital

**DOI:** 10.22034/cmm.2025.345307.1583

**Published:** 2024-12-31

**Authors:** Hoang Dinh Canh, Ngu Thi Tham, Que Anh Tram, Cao Ba Loi, Le Tran-Anh

**Affiliations:** 1 National Institute of Malariology, Parasitology and Entomology, Nam Tu Liem, Ha Noi, Vietnam; 2 Thai Thuong Hoang Hospital, Vinh, Nghe An, Vietnam; 3 The Tropical Diseases Center, Nghe An General Friendship Hospital, Vinh, Nghe An, Vietnam; 4 Scientific and Training Management Department, National Institute of Malariology, Parasitology and Entomology, Nam Tu Liem, Ha Noi, Vietnam; 5 Department of Parasitology, Vietnam Military Medical University, Vietnam

**Keywords:** Oral candidiasis, HIV, RFLP, Vietnam

## Abstract

**Background and Purpose:**

Oral candidiasis (OC) is a common condition in HIV-infected individuals. This study aimed to identify the prevalence, associated factors, and causative agents of OC among HIV-infected patients in a general hospital in Vietnam.

**Materials and Methods:**

The study involved 393 HIV-infected individuals treated at The Tropical Diseases Center, Nghe An General Friendship Hospital, Vinh, Nghe An, Vietnam from January 2022 to May 2024. The sample collected from the buccal mucosa was seeded onto CHROMagar^TM^ *Candida* to isolate and identify the causative yeasts. Molecular identification was performed with restriction fragment length polymorphism assay using MspI restriction enzyme and sequencing of the internal transcribed spacer (ITS) region

**Results:**

The prevalence of OC was 10.7% (95% confidence interval 7.6 – 13.8). Patients with late WHO HIV clinical stage, poorer hygienic condition, or use of prosthetic were at a higher risk of OC. Ten yeast species were isolated, and 10 (23.8%) patients carried more than one type of yeast species. Out of 54 obtained isolates, *Candida albicans* comprised the most (62.9% isolates and 80.9% patients), followed by *C. tropicalis* (16.4% and 21.4% respectively). Overall, 27 patients (64.3%) were infected with *C. albicans*, and 15 patients (35.7%) were infected with non- *albicans Candida*, alone or in combination with *C. albicans.*

**Conclusion:**

The prevalence of OC in HIV-infected patients was low and associated with both systemic and local factors. *C. albicans* was still the most common species but non- *albicans Candida* or coexistence of *Candida* species is frequent.

## Introduction

Human immunodeficiency virus (HIV) is still a major global health problem with about 40 million people living with HIV worldwide at the end of 2023 [ 1 ]. In humans, HIV infects and kills many types of immune cells, resulting in the suppression of cell-mediated immunity [ 2 ]. Consequently, HIV-infected individuals are at a higher risk of opportunistic infection. Among them, oral candidiasis (OC) is a common condition which can affect three-fourths to fourth-fifths of HIV-infected individuals [ 3 - 5 ]. This lesion has been found to be the first sign suggestive of HIV infection [ 6 ] and the only oral lesion that is significantly predictive of immunosuppression in HIV-infected patients [ 5 ]. In addition, the co-infection of HIV and *Candida* may be a factor that enhances the occurrence and progression of other more severe diseases in HIV-infected individuals [ 7 ], specifically the occurrence of esophageal candidiasis, a defining AIDS disease [ 8 ]. Thus, the prevention and treatment of OC is important to maintain the quality of life and reduce the HIV-associated morbidity for the infected individuals. 

In literature, the prevalence of this condition varies significantly across observations, from 5.8 98.3% [9.7]. The high variations in prevalence may be due to the difference in associated factors [ 3 , 9 - 10 ]. The most common responsible agent for OC is *Candida albicans* [ 11 , 12 ], Recently, the emerging non- *albicans Candida* species that usually show more resistance to antifungals than *C. albicans* have been reported [ 13 ]. So, the updated information on prevalence, associated factors, and species distribution in different subpopulations is important for epidemiological, prevention and treatment reasons.

 Vietnam is a Southeast Asian country with a population of about 100 million [ 14 ] and nearly 250,000 adults and children living with HIV [ 15 ]. In a review of studies published during the period January 1995 to August 2014, Vietnam is one of the 12 countries that contribute to 90% of new HIV infections in Asia [ 4 ]. However, the data on OC, specifically the causative agents is limited [ 4 , 16 , 14 ]. Therefore, this study aimed to identify the prevalence, associated factors, and causative agents of OC among HIV-infected patients in a general hospital in Vietnam. 

## Materials and methods

### Participants and study setting

The study was conducted at The Tropical Diseases Center, Nghe An General Friendship Hospital, Vietnam from January 2022 to May 2024. All patients who were infected with HIV and agreed to take part in the current study were recruited. A structured questionnaire was used to obtain information on demographic characteristics (age, gender), socioeconomic status (residence, monthly income, level of education), and unhealthy habits (smoking, harmful consumption of alcohol) of each participant. Data on clinical history including HIV diagnosis date, recent HIV viral load results, use of antiretroviral and antibiotic drugs, and possible associated diseases were collected from participants’ medical records.

### Sample size

A standard formula ($n=z_{2}^{\mathrm{1-\alpha /2}}\mathrm{p(1-p)}/d^{2}$) was used to calculate the sample size. With the estimated proportion (p) of 0.5 [ 17 ], the confidence interval (CI) at 95% and absolute precision (d) of 5%, the sample size required for the study (n) was 384.

### Sample collection, culture, and identification of Candida species

The samples were collected using sterile swabs from lesions in the participants' oral mucosa. The obtained samples were inoculated on CHROMagar^TM^ Candida slants containing gentamicin (0.02 mg/mL) at room temperature under aerobic conditions. Based on the colour and morphology of the colonies, the yeast species were presumptively identified according to the description of the manufacturer. 

### Molecular identification

Pure cultures of the isolated strains were homogenized in 100 μL of sterile water (Corning, USA) and incubated with sorbitol buffer (1M sorbitol, 100 mM EDTA, 14 mM β-mercaptoethanol) and 200UI lyticase (Sigma-Aldrich, USA) for 60 min at 30°C. After disrupting the fungal cell, the genomic DNA was extracted using the QIAamp DNA Mini Kit (Cat.No51304, QIAGEN, Hilden, Germany), following the manufacturer’s instruction. 

The PCR amplification of ITS1-5.8S-ITS2 rDNA regions was performed using ITS1 (5'-TCC GTA GGT GAA CCT GCG G-3') and ITS4 (5'- TCC TCC GCT TAT TGA TAT GC-3') (Integrated DNA Technologies, USA) [ 18 ].

RFLP was performed in tubes containing 5 μl of PCR product, 1 μL of *Msp1*(10UI), 1 μL of 10 × Tango buffer (Thermo Fisher Scientific, USA) and 9 μl of distilled water and incubated at 37°C for 3 hours. Six 6μl of each PCR and digestion product was added 1 μl of loading dye buffer and then electrophoresed on 2% agarose gel in 1× TBE buffer for about 2.0 h at 90V. After staining with 0.5 μg/ml of ethidium bromide the products were visualized on UV illumination (UVP, Canada). The size of each band was determined by a 100 bp ladder (Thermo Fisher Scientific, USA). The species identification of the strain was performed based on the size of PCR and RFLP products according to the method described by Mirhendi et al. [ 19 ].

Represent isolates of groups having consistent result of species identification, isolates that could not be identified by the above two methods, or with inconsistent results were further analyzed by sequencing the internal transcribed spacer (ITS) regions. The obtained nucleotides were then compared to database sequences in GenBank to make species identification. 

### Definition

The clinical stage of the patients was classified according to WHO criteria [ 20 ]. Harmful consumption of alcohol was applied for males drinking more than two drinks per day or for females drinking more than one drink per day [ 21 ].

### Statistics

Data were analyzed using SPSS software version 22.0. The categorical data was described by case number (n) and percentage while the continuous data was present as mean and standardization deviations (SD). Univariate analysis was used to evaluate the association between a dependent variable and the presence of OC. The variables having significant relationships in univariate analysis were subjected to multivariate analysis. The p values (two-tailed) < 0.05 were considered statistically significant. 

## Results

Individual data of 393 participants including 231 (58.8%) males and 162 (41.2%) females are summarized in Table 1. The age of participants ranged from 18 to 74 years with a mean of 44.02 ± 8.17 years. Almost all the patients (96.9%) were on antiretroviral therapy (ART) with a median time of 106 months (25th–75th percentile: 90–145 months). Most of the patients were at the WHO I clinical stage and had low HIV load. 

**Table 1:** Baseline characteristics of the participants (n= 393)

Variable		N (%) or mean ± SD	
Age	< 40	103	26.2
	40 – 49	215	54.7
	≥50	75	19.1
	Mean ± SD	44.02 ± 8.17	
Gender	Male	231	58.8
	Female	162	41.2
Income (million VND)	≤5	102	25.95
	>5 - 10	254	64.63
	> 10	37	9.41
	Median	7	
Level of education	Primary school	14	3.6
	Secondary school	147	37.4
	Tertiary	221	56.2
	Higher level	11	2.8
Residence	Urban	170	43.3
	Rural	192	48.9
	Mountain	31	7.9
Smoke		89	22.6
Alcohol		32	8.1
Oral hygene	Brushing once daily	36	9.2
	More than once daily	357	90.8
Wearing prothesis		63	16.0
Comorbidity		88	29.01
Antibiotic usage (≥ 7 days)		47	11.96
Rout of infection	Injection	180	45.80
	Sex	265	67.43
HIV duration (year)	Less than 1 year	19	4.83
	1 up to less than 5	20	5.09
	5 up to less than 10	156	39.69
	10 up to less than 15	139	35.37
	15 years or more	59	15.01
WHO clinical stage	I	362	92.1
	II	18	4.6
	III	11	2.8
	IV	2	0.5
HIV load (copies/mL)	<20	321	81.68
	20 – 1000	56	14.25
	> 1000	16	4.07

VND: Vietnam dong (Vietnam money).

There were 42 (10.7%, 95% CI 7.6 – 13.8) HIV-infected patients having OC. Results of the univariate analysis showed a significantly higher prevalence of OC in participants who had lower income or level of education, lived in mountainous regions, or had unhealthy habits (smoking, consumption of alcohol). Patients with poor oral hygiene and wearing prostheses were more likely to be infected with yeast in the oral cavity (p< 0.05). Comorbidity, antibiotic usage ≤ 7 days, later WHO HIV clinical stage and longer HIV duration were medical factors that were statistically associated with OC in the sample (Table 2).

**Table 2:** Prevalence of oral candidiasis and associated factors on univariate analysis

		No test	No positive (%)	OR (95% CI)	p value
**Age group**	< 40	103	11 (10.7)	NA	0.627
	40 – 49	215	22 (10.2)		
	≥50	75	9 (12.0)		
**Gender**	Male	203	28 (12.1)	1.458 (0.742 - 2.866)	0.321
	Female	148	14 (8.6)		
**Income (million VND)**	≤ 7	215	30 (14.0)	2.243 (1.112 - 4.524)	0.022*
	> 7	178	12 (6.7)		
**Residence**	Urban	170	13 (7.6)	NA	0.042*
	Rural	192	22 (11.5)		
	Mountain	31	7 (22.6)		
**Education**	Lower	136	25 (15.5)	2.325 (1.211 – 4.464)	0.012*
	Tertiary or higher	215	17 (7.3)		
**Smoke**	Yes	89	21 (23.6)	4.162 (2.150 -8.054)	<0.001*
	No	304	21 (6.9)		
**Alcohol**	Yes	32	9 (28.1)	3.889 (1.663- 9.097)	<0.001*
	No	375	33 (10.7)		
**Oral hygiene**	Brushing once daily	36	23 (63.9)	31.474 (13.831 - 71.621)	<0.001*
	More than once daily	338	19 (5.3)		
**Prothesis**	Yes	63	14 (22.2)	3.082 (1.517- 6.262)	0.003*
	No	330	28 (8.5)		
**Comorbidity**	Yes	88	26 (29.5)	7.575 (3.835- 14.959)	<0.001*
	No	315	16 (5.2)		
**Antibiotic usage (≥ 7 days)**	Yes	28	19 (67.9)	31.391 (12.782- 77.093)	<0.001*
	No	378	23 (6.3)		
**WHO HIV clinical stage**	II-IV	31	30 (96.8)	875.000 (109.995 - 6960.550)	<0.001*
	I	362	12 (3.3)		
**HIV duration (years)**	< 5	39	11 (28.2)	NA	<0.001*
	5 – 10	156	13 (8.3)		
	> 10	198	18 (9.1)		
**HIV viral load (copies/mL)**	<20	321	30 (9.3)	0.515 (0.250 - 1.064)	0.089
	≥ 20	72	12 (16.7)		
**Rout of infection**	Inject	158	22 (13.9)	1.739 (0.915 -3.306)	0.098
	Sex	245	20 (8.5)		
**Total**		393	42 (10.7)	(7.6 - 13.8)	

CI = Confidence Interval, P Value is Significant at 0.05 level; NA = Not Applicable; OR = Odds Ratio; CI = Confidence Interval;

* = statistically significant;

+ = Chi square by trend

Results of a multivariate analysis revealed 3 factors statistically associated with oral candidiasis among HIV patients. The factors included wearing prostheses, poor oral hygiene and WHO clinical stage II-IV (Table 3). 

**Table 3:** Results of multivariate analysis of factors associated with oral candidiasis

	95.0% C.I. for aOR			
	p	aOR	Lower	Upper
Income*	0.105	3.538	0.769	16.286
**Educational level†**	0.397	0.474	0.085	2.661
**Smoke**	0.477	1.806	0.354	9.209
**Alcohol**	0.272	0.322	0.042	2.439
**Prothesis**	0.001#	11.012	2.712	44.711
**Comorbidity**	0.736	1.337	0.248	7.206
**Residence‡**	0.245	0.117	0.003	4.360
**Antibiotic use**	0.572	2.400	0.115	50.087
**Oral hygiene§**	0.000#	32.464	5.567	189.330
**HIV stage||**	0.000#	1.109E3	61.312	2.007E4
**HIV duration (years) ¶**	0.384	2.467	0.322	18.873

a OR = Adjusted Odds Ratio,

* ≤ 7 vs > 7 million Vietnam dong,

† Primary and secondary school vs tertiary or higher level,

‡ People living in mountainous regions vs. those in urban or rural areas,

§ Comparing between participants who brush mouth once daily vs. brush mouth more than twice daily,

|| Stage II-IV vs. stage I,

¶ ≥ 10 vs < 10 years,

# Significant at 0.05 level

There were 54 morphologically distinct colonies isolated from positive cultures of 42 patients. There were ten species identiﬁed, and most of the patients (32, 76.2%) carried one type of yeast species. There were 8 patients (19.0%) had two different species (three had *C. albicans* and *C. tropicalis*; the five other had *C. albicans* and *C. glabrata*, *C. albicans* and *Kodamaea ohmeri*, *C. tropicalis* and *C. dubliniensis*, *C. krusei* and *C. dubliniensis*, *C. parapsilosis* and *C. metapsilosis*) and 2 (4.8%) had three species (one had *C. albicans*, *C. tropicalis*, Meyerozyma caribbica and the other carried *C. albicans*, *C. mesorugosa* and *K. ohmeri*). Totally, out of 54 obtained isolates, *C. albicans* comprised of 34 (62.96% isolates and 80.95% patients) and *C. tropicalis* comprised 9 (16.36% and 21.43% respectively). *C. dubliniensis* and *C. mesorugosa* comprised 2 isolates each while *C. glabrata*, *C. krusei*, *C. parapsilosis*, *C. metapsilosis* and *M. caribbica* accounted for 1 isolate. Overall, 27 patients (64.3%) were infected with *C. albicans*, and 15 patients (35.7%) were infected with non- *albicans Candida*, alone or in combination with *C. albicans* (Table 4).

**Table 4:** The distribution of yeast species

Genus	Species	n	Isolation rate by patients, % (42)	Isolation rate by species, %, (54)
***Candida species***	*C. albicans*	34	80.95	62.96
	*C. tropicalis*	9	21.43	16.36
	*C. dubliniensis*	2	4.76	3.70
	*C. mesorugosa*	2	4.76	3.70
	*C. glabrata*	1	2.38	1.82
	*C. krusei*	1	2.38	1.85
	*C. parapsilosis*	1	2.38	1.82
	*C. metapsilosis*	1	2.38	1.82
***Kodamaea***	*K. ohmeri*	2	4.76	3.70
***Meyerozyma***	*M. caribbica*	1	2.38	1.82

## Discussion

### The prevalence

The first aim of the study is to find out the prevalence of OC and its associated factors among HIV-infected patients in a general hospital in Vietnam. The OC prevalence of 10.7% obtained in the current study is comparable to those reported in Uganda (7.6%) [ 12 ], Cameroon (11.0%) [ 22 ], and Taiwan (12%) [ 11 ]. However, this rate is much lower than the prevalence of 37.5% [ 16 ], and 54% previously reported in Vietnam [ 3 ]. This variation may be due to the difference in the participants. The patients involved in previous studies in Vietnam were those having severely compromised immune systems (the median CD4 count was 20 cells/mm3) [ 3 ] or late-stage HIV disease [ 16 ] whereas our sample included all individuals infected with HIV, irrespective of their immunology status or the clinical stage. In addition, almost all the participants in the current study were on ARV therapy which has been shown to decrease the risk of OC in HIV-infected patients [ 23 , 24 ].

### Associated factors

This study found a significant relationship between the clinical stage of HIV, oral hygienic condition, wearing prosthetics and the occurrence of OC (Table 2). The association between the WHO HIV clinical stage and OC have been documented in many studies [ 10 , 25 ]. In a review of this topic, McCarthy et al. (1991) found that the advanced stage was the most important factor associated with OC in HIV-infected patients [ 26 ]. The increased incidence of OC in the late HIV clinical stage suggests the severity of immunosuppression which is responsible for an increasing susceptibility of the patients to opportunistic infections. Nanteza et al. (2014) have proven that OC is the only oral lesion that is significantly predictive of immunosuppression in HIV-infected patients [ 5 ]. Poor oral hygiene [ 27 , 28 ], and wearing prosthetics have been proven as local factors that predispose to oral candidiasis even in otherwise healthy individuals [ 29 ]. In HIV-infected individuals, a study conducted in Cameroon revealed that the incidence of OC in patients who bush mouth once daily is significantly higher compared to those who performed mouth hygiene twice daily (adjusted OR: 2.15; 95% CI: 1.32-3.50, p=0.002) [ 28 ]. Witzel et al. (2012) conducted a study involving 193 HIV-infected patients and demonstrated that the prevalence of OC was significantly higher in patients wearing removable dental prostheses compared to their counterparts (10.7% vs. 3.3%, p = 0.0065) [ 30 ]. This association may have a significant clinical implication in suggesting that hygienic measures should be strengthened to prevent OC in such high-risk patients. According to the Infectious Diseases Society of America, disinfection of dentures is recommended for patients with denture-related candidiasis [ 31 ].

### Species distribution

The second aim of the study is to find out the fungal species responsible for OC in HIV-infected patients. Among ten species identiﬁed in the present study, *C. albicans* was the predominant one (80.95% of the isolates and 62.96% of the patients) which is consistent with previous data [ 11 , 32 ],. On the global scale, the isolation rate of *C. albicans* is on a decreasing trend [ 33 ]. However, the prevalence of *C. albicans* isolated from the oral cavity is still remarkably high, especially in individuals with HIV infection [ 32 ]. 

This dominance may have resulted from many virulent factors of *C. albicans* such as their capacity for adherence to host tissues, biofilm formation, and secretion of hydrolytic enzymes [ 34 ]. The second most frequent agent in our sample was *C. tropicalis* (21.43% of the isolates and 16.36% of the patients), the agent considered the second most virulent *Candida* species, behind *C. albicans* [ 35 ]. In Vietnam, there have been studies showing that *C. tropicalis* is the most frequent agent isolated from patients with candidemia [ 36 ] or major burns [ 37 ]. The other *Candida* species isolated in the present study such as *C. glabrata*, *C. krusei*, *C. parapsilosis* have been commonly isolated in the oral cavity of healthy as well as HIV-infected populations [ 11 , 12 ]. *Kodamaea (Pichia) ohmeri* was also detected in the HIV-infected population [ 13 ]. *Meyerozyma caribbica* is a species belonging to *Meyerozyma guilliermondii* complex and has been isolated from patients with candidemia [ 38 ]. The coexistence of two or more species in 10 (23.8%) patients in the current study has also been reported elsewhere [ 12 , 25 ]. Earlier studies reveal that the coexistence of more than one *Candida* species in the oral cavity is higher in HIV-infected or immunosuppressed populations than in the control group [ 39 ]. The high rate of *C. albicans* infection in this study (64.3% of patients) may have a practical implication because of the low antifungal resistance of this yeast [ 33 ]. However, the fact that more than one-third of patients (35.7%) infected with non- *albicans Candida*, alone or in combination with *C. albicans*, need to be paid much attention. On the global and country scale, non- *albicans Candida* has been found to have a higher rate of antifungal resistance, especially against fluconazole– the first-line pharmacologic treatment option for OC in HIV-infected patients [ 33 , 40 ], in comparison with *C. albicans* [ 33 , 37 ].

This study has some limitations. The cross-sectional design of the study makes it impossible to find the true understanding of causal pathways; so, the risk factors could only be commented on as being associated with OC. In addition, the study is potentially affected by recall bias, specifically for items such as unhealthy habits or medical data of the participants.

## Conclusion

In summary, the results of this study suggest a low prevalence of oral candidiasis in patients infected with HIV (10.7%). The late stage of HIV infection and the local condition of the oral cavity are factors predicting the occurrence of oral candidiasis. *Candida albicans* has been the most predominant species, and the coexistence of more than one *Candida* species was frequent. Better hygienic measures may reduce the frequency of HIV- associated oral candidiasis, especially for those with late stages of HIV or wearing dentures. 
